# Universal Stress Proteins as New Targets for Environmental and Therapeutic Interventions of Schistosomiasis

**DOI:** 10.3390/ijerph13100972

**Published:** 2016-09-30

**Authors:** Priscilla Masamba, Abiola Fatimah Adenowo, Babatunji Emmanuel Oyinloye, Abidemi Paul Kappo

**Affiliations:** 1 Biotechnology and Structural Biochemistry (BSB) Group, Department of Biochemistry and Microbiology, University of Zululand, KwaDlangezwa 3886, South Africa; presh4u@rocketmail.com (P.M.); afaatimah@yahoo.com (A.F.A.); tunji4reele@yahoo.com (B.E.O.); 2 Department of Biochemistry, Afe Babalola University, PMB 5454, Ado-Ekiti 360001, Nigeria

**Keywords:** cercariae, *Schistosoma mansoni*, schistosomiasis, schistosomulae, praziquantel, universal stress proteins

## Abstract

In spite of various control measures and eradication methods that have been in progress, schistosomiasis still prevails as one of the most prevalent debilitating parasitic diseases, typically affecting the poor and the underprivileged that are predominantly concentrated in sub-Saharan Africa. The parasitic schistosome blood fluke responsible for causing the disease completes its complex developmental cycle in two hosts: humans and freshwater snails, where they physically undergo gross modifications to endure the different conditions associated with each host. Just like any other organism, the worm possesses mechanisms that help them respond to environmental insults. It has been hypothesized that a special class of proteins known as Universal Stress Proteins (USPs) are up-regulated during sudden environmental changes, thus assisting the worm to tolerate the unfavourable conditions associated with its developmental cycle. The position of praziquantel as the drug of choice against all schistosome infections has been deemed vulnerable due to mounting concerns over drug pressure and so the need for alternative treatment is now a matter of urgency. Therefore, this review seeks to explore the associations and possible roles of USPs in schistosomiasis as well as the functioning of these proteins in the schistosomulae stage in order to develop new therapeutic interventions against this disease.

## 1. Introduction

In comparison to equally virulent diseases such as tuberculosis, malaria and HIV/AIDS, schistosomiasis still persists as one of the most neglected, prevalent and parasitic diseases of poverty [1]. Its high degree of morbidity and consequential disabling effects are still severely underestimated though millions of lives are affected [2]. The disease is endemic in more than 76 countries, affecting more than 200 million people with a mortality rate estimated to be more than 250,000 deaths per annum in sub-Saharan Africa alone [3,4]. Additionally, it has been documented that more than 700 million people are still at risk of infection [3]. Countries such as Venezuela, Brazil, Nigeria and the Democratic Republic of Congo fall under those most hit with the disease burden [5,6].

The disease mostly affects poor rural dwellers who are in constant and regular contact with open water sources and largely dependent on agricultural and irrigation activities. Poverty is one of the key underlying factors that form a vicious “poverty-trap” cycle in developing countries. There is a clear link between schistosomiasis and poverty due to the fact that the disease mostly affects individuals during their most productive years. The effect of schistosomiasis on agricultural productivity, pregnancy outcome, child development and most importantly, the economy has been documented as stimulating poverty, which in turn increases risk of infection [6,7,8]. Poverty and underdevelopment in areas with limited healthcare resources and personnel compound the problem. With insufficient education, unchanged habits and inadequate water supply from proper sources, re-infection rates still remain high. About 6–13.5 million disability-adjusted life-years (DALYs) of healthy lives are lost globally and therefore reducing transmission, infection and re-infection is quickly becoming a control priority [9].

**Figure 1:**
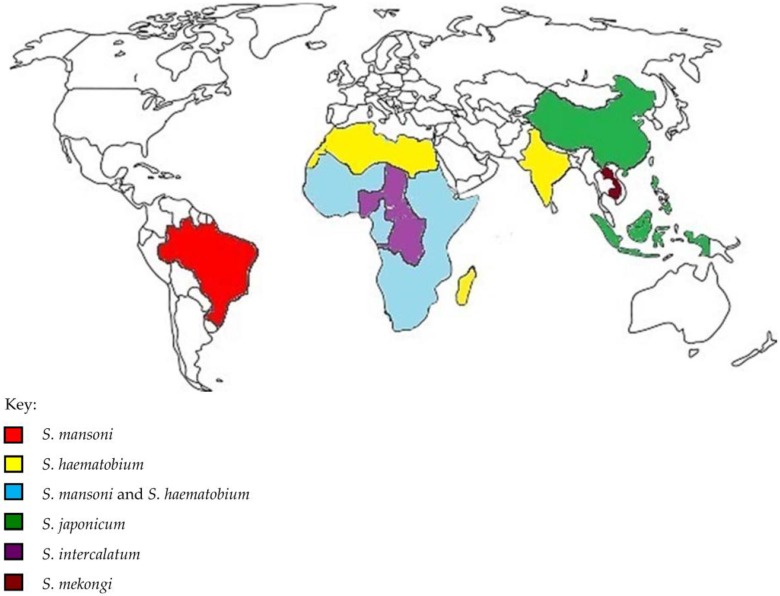
Geographical distribution of the different schistosome species. Each of these species has their own geographic pattern: *Schistosoma mansoni* (*S. mansoni*) infection mostly occurring in sub-Saharan Africa, the Middle East, South America; *Schistosoma intercalatum* (*S. intercalatum*) in Congo, Nigeria, Chad and Central African Republic; *Schistosoma haematobium* (*S. haematobium*) is mostly prominent in sub-Saharan Africa, North Africa, Middle East, India and the islands of Madagascar and Mauritius; *Schistosoma japonicum* (*S. japonicum*) is endemic to Asian countries only such as China, Philippines and Indonesia and finally *Schistosoma mekongi* (*S. mekongi*) is found exclusively in Laos and Cambodia.

*Schistosoma mansoni* (*S. mansoni*), *Schistosoma haematobium* (*S. haematobium*) and *Schistosoma japonicum* (*S. japonicum*) are the three main species mostly responsible for parasitizing humans while *Schistosoma mekongi* (*S. mekongi*) and *Schistosoma intercalatum* (*S. intercalatum*) are more rare and confined to only a few countries [4,10]. Each of these species has their own geographic pattern: *S. mansoni* infection mostly occurring in sub-Saharan Africa, the Middle East, South America; *S. intercalatum* in Congo, Nigeria, Chad and Central African Republic; *S. haematobium* is mostly prominent in sub-Saharan Africa, North Africa, Middle East, India and the islands of Madagascar and Mauritius; *S. japonicum* is endemic to Asian countries only such as China, Philippines and Indonesia and finally *S. mekongi* is found exclusively in Laos and Cambodia (Figure 1) [11,12]. Japan, Algeria, Morocco and Tunisia have successfully eliminated the disease, while China and Egypt have effectively implemented control programmes, all showing that elimination is a target that can be achieved with the use of preventive chemotherapy and transmission control tools [13]. However, the disease has now spread to regions that were non-endemic before and it is feared the building of dams, development of irrigation systems and global warming could further increase schistosomiasis transmission [1]. The development of water resources have the advantages of increasing crop yields, dealing with water scarcity, generating hydropower and accumulating export earnings, but schistosomiasis easily detects ecological make-overs due to its wide distribution and changing infection rates [14]. Other factors have also complemented the problem such as the presence of intermediate snail species. Urinary schistosomiasis was last detected in Europe in the 1950s. However, the presence of *S. heamatobium* freshwater snails such as *Bulinus contortus*, *Bulinus truncatus* and *Planorbarius metidjensis* in Portugal, Spain and especially in Corsica France (particularly by the Cavu River) could very well cause the re-emergence of the disease in Europe [15]. This also substantiates the suggestion that schistosomiasis not only has the ability to persist locally but can also spread from Africa to countries of higher latitudes [16].

**Figure 2:**
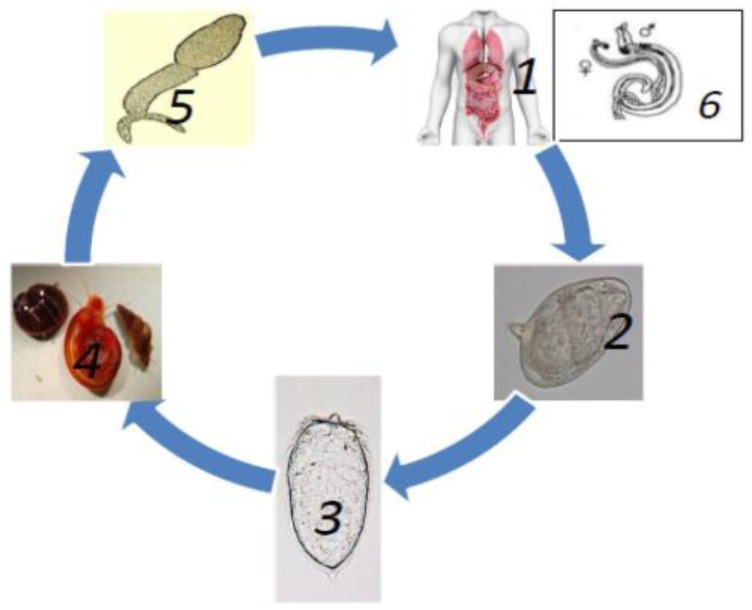
Life cycle of Schistosomes. **1** & **2**: Eggs excreted, either in the urine (in the case of *S. haematobium*) and/or faeces (depending on the species) of infected individuals, begin the devastating cycle and eggs may stay viable for up to 7 days [4,10]. Only then do the eggs hatch in fresh water to become **3**: miracidia under the most favourable environmental conditions [3]. With the help of light and chemical stimuli, the miracidia burrow the tissue of a **4**: freshwater snail. Production of sporocysts and **5**: cecariae occur asexually over a period of 4–6 weeks and are released from the snail. The cercariae have forked or bifurcated tails and embryonic suckers and swim around until they come into contact with the human skin [4,18]. Thereafter, they shed their tails whilst simultaneously penetrating the epidermis to develop into larval forms called schistosomulae which remain in the skin for 48 h [3]. They then circulate the hepatic portal system via the bloodstream to mature into **6**: adults [19]. The worms then mate 28–35 days after infection, producing about 100–300 eggs per day (female *S. mansoni* and *S. haematobium* female worms) and 500–3500 eggs produced daily by *S. japonicum* worms which are released from the human host via defecation, thereby continuing the destructive cycle [20,21]. At this point, the disease seems species-dependent in that adults from *S. mansoni* and *S. japonicum* dwell in the mesenteric venous plexus causing hepatic and intestinal schistosomiasis; while adults belonging to *S. haemotobium* cause urinary schistosomiasis and reside in the perivesical venous plexus [22]. The worms are characterised by having a blind digestive tract, advanced neural, excretory and reproductive organs, two ending suckers and a greyish-white tegument that covers the 7–20 mm body of the worm [3,22]. Females reside in the gyneacophoric canal of the male and together form a nematode-like appearance [5,23].

Throughout their complex lifecycle (Figure 2), schistosomes face a number of hostile and unfriendly environments, transitioning between intra-mammalian, aquatic and snail stages to develop into full maturity [17]. Despite the harsh conditions they are challenged with, the worms have the ability to withstand all these conditions and evade different defense systems they encounter. Various complications, which have been associated with the disease, then result from eggs produced by females, which become trapped in host tissues. It is with this background that this review suggests that Universal Stress Proteins (USPs) have possible roles in *Schistosoma* ecology and therefore, seeks to explore the associations and possible roles of USPs in schistosomiasis in order to develop new environmental and therapeutic interventions against this prevalent debilitating parasitic disease.

## 2. Anti-Schistosomal Drugs

Originally, schistosomiasis control was tackled by elimination of snails until 1984 when chemotherapy was suggested by the WHO Expert Committee [18]. Presently, the likes of praziquantel, oxamniquine and artemisinin are considered as defence systems against the disease. However, oxamniquine does not treat all schistosome infections, its target is only limited to *S. mansoni* adult males, with less action against female and juvenile worms [23]. Use of the drug is mainly restricted to Brazil and South American countries but has been replaced by praziquantel as the principal drug and main line of treatment against schistosomiasis since the mid-2000s [24,25]. Artemisinin is an antimalarial drug which when used with artesunate and artemether combined has been shown to exhibit anti-schistosomal activity against juvenile worms [22]. However, the development of drug resistance in endemic areas against the *Plasmodium* strain has caused a bit of concern.

Discovered in the 1970s, praziquantel (PZQ) is a pyrazinoisoquinole antihelminthic that stands as one of the very few anti-schistosomal drugs available commercially with few known side effects [26]. The drug has been in use for more than 30 years and stands as the most efficient chemotherapeutic drug effective against all forms of schistosome infection. The drug is generally well-tolerated with mild common side effects such as abdominal pains, dizziness, weakness and headache. However, there have been mounting worries over the line of treatment by this drug [27].

As reported by Aragon et al. and several other authors, PZQ is not effective against all stages involved in the life cycle of the worm and fails to kill immature worms 3–4 weeks post-infection [24,28,29,30]. The drug seems stage-dependent, only effective on 1–2 weeks old larvae and 5 weeks and older adult worms [23]. Additionally, the mode of action still remains obscure despite decades of research [31]. So far, it has been suggested that antigens on the surface of the parasite become exposed due to disruption of the tegument caused by the drug. Rapid contraction of the muscle occurs (which is also associated with antigen exposure) as well as muscle paralysis accompanied with a rapid influx of calcium ions, a slower influx of sodium ions and a decreased influx of potassium ions [26,31]. All these responses are thought to be linked to the disruption of calcium homeostasis. An unusual variant β subtype Ca^2+^ channel with unusual structural and functional properties that is especially F-sensitive is thought to cause this calcium influx [31].

Most importantly, reports of drug resistance to praziquantel due to drug pressure have now put the health and scientific community on edge. Despite countless treatments made with the drug, the “KCW” isolate found in a patient from Kisumu in Kenya had grown remarkably resistant to praziquantel and even developed a heritable trait that maintained resistance to the drug more than six generations later [32]. A Senegalese isolate also showed less susceptibility to praziquantel as compared to other *S. mansoni* worms from Puerto Rico and Kenya [27]. In Egypt, by the Nile Delta region, reports of *S. mansoni* strains growing resistance to praziquantel were reported after villagers were treated with the drug three times successively 6–8 weeks apart but still continued to shed viable eggs in their stool [33]. These villagers were than revisited and retreated 7–8 years later with the same drug but 4% were still left uncured after as single dose [34]. Apart from field isolates, experimentally induced isolates have also been reported. Coeli et al. observed and reported a 3-fold increase during seven parasite generations amongst mouse models treated with increasing doses of PZQ [35]. Fallon and co-workers were also able to mention 2 isolates in two separate laboratories, where both exhibited PZQ-resistant behavior [27]. More recent experimentally-induced PZQ resistance has been reported by Couto et al. where infected *Biomphalaria glabrata* snails underwent PZQ treatment and subsequent cercarieae produced (LE-PZQ isolates) were used to infect mice which also underwent PZQ treatment. Results showed accumulated resistance and reduced susceptibility to PZQ [36].

With this disease still prevalent in many countries and even exhibiting signs of resistance on PZQ, which at the current moment is the most reliable drug, depending on this one line of treatment and the very few other available therapies is quite unreasonable. Targeting the parasitic lifecycle and its different developmental stages could well provide a lasting solution. However, one needs to also consider the abiotic and biotic stresses associated with them.

## 3. Parasitic Lifecycle and Stress

The environment undergoes constant change spontaneously and this may have severe effects on the lifecycle of the *Schistosoma* worm. The intermediate hosts are also equipped to defend themselves internally against infective parasitic stages. Such conditions may manifest or be grouped in either of two forms: biotic or abiotic stress.

### 3.1. Abiotic Stress

The eggs, miracidia and cercariae are exposed to the freshwater environment and have to endure the stress conditions associated with it [17]. The range in water temperature has important bearing on the hatching of eggs and shedding of cercariae. Warming of water may decrease or increase the number of snails, especially in endemic areas, which in turn has an effect on snails being infected with cercariae [37]. Eggs hatch between 10 °C and 30 °C even though temperatures vary between 30 °C and 38 °C in a period of 17 days based on the time miracidia first penetrate snail tissue and snails release cercariae [38]. Hatching of the eggs is also dependent on osmotic pressure and light. The speed and movement of water, either rapidly moving or standing water can also affect the ability of miracidia to effectively locate and infect snails and it has been reported that free-flowing water increases the chances of miracidia to come into contact with their intermediate snail hosts [39].

### 3.2. Biotic Stress

Miracidia penetrate the freshwater *Biomphalaria* intermediate host, usually at the base of its antennae and cephalopodal mass to mature into primary sporocysts, later spawning into secondary sporocysts that migrate from the muscles of the cephalopodal region to the digestive glands or hepatopancreas of the snail [40]. From there, the structure changes to form cercariae. The internal defence system (IDS) of the snail consists of hemocytes, which are responsible for killing these larval stages [41]. Additionally, in the hemolymph are soluble elements that produce toxic substances that recognize the parasite as well as activate the hemocytes [41]. Resistance or susceptibility of the parasite to the snail host is mostly controlled by genetic factors [42]. However, new evidence has suggested that snail susceptibility or resistance for schistosome infection may also depend on epigenetic factors. It has already been previously established that indicators of stress such as Hsp70 and Hsp90 proteins indicate stress levels in susceptible (NMRI) rather than in resistant (BS-90) snails. However, four generations of BS-90 resistant snails grown and maintained at a mild heat shock temperature of 32 °C lost their resistance when returned to room temperature (25 °C), shedding cercariae 4 weeks after exposure to the environment [16]. More so, the snails exhibited greater expression of Hsp70 protein. Additionally, the parasites also need to be compatible with the snails they encounter. Compatibility is heritable, either for the resistant or susceptible snail or in the parasite’s ability to infect an intermediate snail host. It may also be strain specific in that snails susceptible to a particular schistosome strain might not be so susceptible to another strain [43]. Theron and co-workers were able to show that among five *B. glabrata* and five *S. mansoni* strains, some snails had greater ability in recognizing parasites and producing ROS thus having a more efficient and effective defence system [43]. Other biotic factors directly or indirectly having an impact on these schistosomal stages include natural predators like invertebrates and fish which may reduce miracidia population by feeding on them [38].

The human host also has defence systems set in place to protect it from foreign harmful agents. Cercariae penetrate the skin of a human host, simultaneously shedding their tails to transform into schistosomulae. An antibody-dependent cell-mediated cytotoxic (ADCC) response is responsible for eliminating this parasitic stage by the production of reactive oxygen species such as superoxide radical anion and hydroxyl radicals [44]. Additionally, the release of cytotoxic proteins to produce nitric oxide may also contribute to the removal of schistosomulae [44]. Infection with schistosomiasis stimulates a T_H_1 (which is the dominant response) and T_H_2 response which provides immunity against the parasite and this has been shown based on experimental mouse models. The T_H_1 response is up-regulated during the migration of immature worms during the first few weeks of infection and later down-regulates to allow for the expression of the T_H_2 response during mating and egg production [45]. Cytokines with immunoregulatory functions like IL-10 are generated after cercariae exposure along with other pro-inflammatory cytokines like IL-12, IL-1β, MIP1α, IL-6 and TNFα [46]. Responses are more T_H_2-associated in the event of re-infection. IgG4 has been shown to act as an antibody by blocking IgE action thereby decreasing susceptibility to re-infection while in contrast; IgE receptors have also been associated with protecting the host against re-infection [47].

In short, major morphological transitions have to take place between the three different and distinct environments. The miracidium larval stage has to tolerate freshwater aquatic environments. The sporocysts, parasites of the intermediate snail host, reside in a freshwater environment while the cercariae habituate an aquatic environment before finding a human host. Finally, the parasite develops in a different setting of a well-defended warm-blooded mammalian host to develop into two different sexual stages to produce eggs and continue the cycle.

Therefore, the question to be asked is this: how then does the schistosome parasite have the ability to withstand the inimical conditions associated with each hostile environment? Mbah and co-workers suggested that amid other cellular and molecular mechanisms that may also have an input in their survival, the presence of Universal Stress Proteins (USPs) could be an important factor or an interesting drug target that could be developed for therapeutic interventions to take place in the developmental cycle of the parasite [17].

## 4. Universal Stress Proteins, Mode of Action and Factors that Induce and Regulate Their Activity

An environment undergoes constant change, which may either be good, bad, sudden or seasonal. Organisms should therefore be equipped with tactics to survive and respond to these environmental changes. Environmental stress may appear in various forms, which can result in the denaturation of proteins of an organism eventually leading to severe harm or even death [17]. Various molecular and response mechanisms may be responsible for this quality in many organisms such as the expression of Heat shock proteins (HSPs). It has been suggested that in schistosomes, the up-regulation of a special class of proteins could enable them to withstand the different and mostly unfavourable environments and different hosts the parasite occupies during their complex developmental cycle called Universal Stress Proteins (USPs) [17].

**Figure 3:**
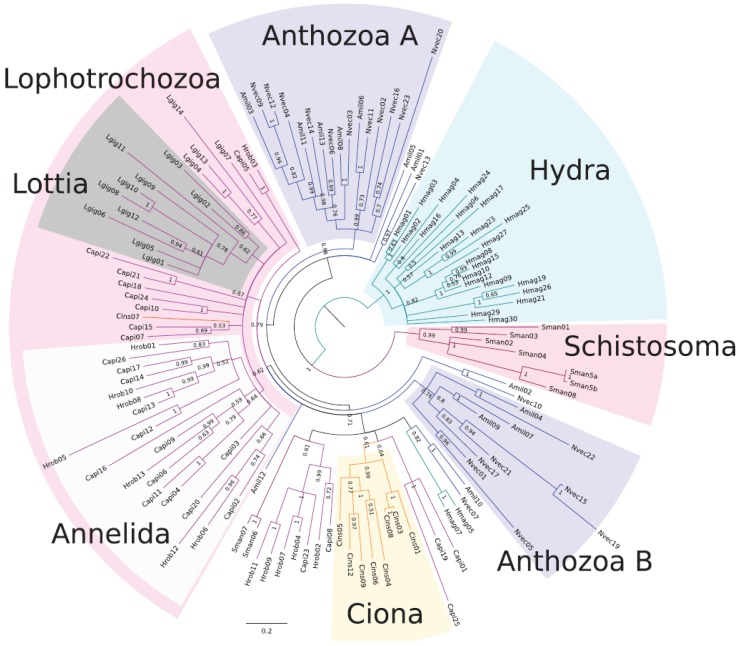
Phylogenetic analysis of USP sequences from animals using maximum likelihood and Bayesian methods. The figure was taken from Foret et al. [49].

USPs have been shown to be present among a diverse group of organisms such as bacteria, protists, fungi and plants (Table 1) [48,49,50,51,52,53,54,55,56,57,58,59], assisting them to tolerate different environmental and adverse conditions such as high salinity, high temperatures, drought and toxic chemicals [49]. Originally, universal stress protein A was first discovered in *Escherichia coli* but lately, genes encoding proteins with the USP domain (Pfam Identifier: PF00582) have recently been shown to be present in the *S. mansoni*, *S. japonicum* and *S. haematobium* species [17,49,60]. This protein is a member of what is known as the “universal stress protein (USP) domain” family, which is a conserved domain, constitutes about 140–160 residues [50]. Foret and co-workers also identified and reported the presence of USPs in metazoans based on EST data encoding sets (Figure 3) [49]. However, many of the gene families have been lost during animal evolution suggesting that either their roles or functions are no longer needed or they have evolved to suit different and specific animals [49]. Majority of the genes are small single domain proteins with some considered ATP-binding.

USPs may exist as small proteins (~14–15 kDa) with a single domain, as larger proteins (~30 kDa) also with a single domain or in a combination with one or two USP domains having functional domains that include, voltage-gated channels, protein kinase domains, antiporters and amino acid permeases [60]. Several duplicates of USPs may exist in certain organisms such as the six Usps present in *Escherichia coli* (UspA, UspC (*yecG*), UspD (*yiiT*), UspE (*ydaA*), UspF (*ynaF*) and UspG (*ybdQ*)) and they may also be encoded by different gene families [52,58]. There are 8 known *S. mansoni* USP genes: (Smp_001000, Smp_001010, Smp_031300, Smp_043120, Smp_076400, Smp_097930, Smp_136870 and Smp_136890) and all have shown conserved sites for Aspartate, Leucine, Glycine, Histidine and Proline which seem to be common functional sites for regulating these USPs [60]. USP proteins are generally categorized into two main groups based on the presence or absence of an ATP binding motif G-2x-G-9x-C(S/T) (which is a molecular mechanism responsible for regulating their functions) [17]. UspAs and those categorized as UspA-like proteins do not bind ATP while UspFGs bind ATP [48]. All 8 *S. mansoni* USP genes contain this motif [60]. ATP binding up-regulates the protein by acting as a donor phosphate and together with guanosine triphosphate (GTP), phosphorylates USPs onto serine, activating the protein and showing that other cellular factors such as ATP contribute to the activity of the protein through phosphorylation. It has been suggested that Usp proteins that bind ATP are part of the ancient HUP (**H**IGH-signature proteins, **U**SP-like domains and **P**P-ATPases) superfamily whose domains contain an α/β core and characterized by 5-stranded parallel β sheets and 4 α helices [53,54].

Another contributing factor is the predicted presence of metallic cations such as magnesium, calcium and zinc. It has been suggested that by binding to the Mg-ATP-binding groove during the phosphorylation process or any response to stress that is dependent on ATP, Mg^2+^ could play a vital role in protein functional efficiency [17]. The role of calcium in certain developmental stages and its association with the mechanism of action of PZQ has been suggested while work is ongoing to establish the transcriptional response of *Schistosoma* USP genes to PZQ which could eventually lead to new schistosome control interventions [17].

## 5. Role of USPs in Disease Progression

Jolly and co-workers reported the up-regulation of 1154 genes with gene expression transitions observed between the sporocysts, cercariae and adult stages of *S. mansoni* [61]. Out of these genes, 406 of them are said to currently have unknown functions. Factors like genes involved in protein synthesis (such as heat shock genes), apoptosis, ubiquitination, and proteolysis in the sporocyst stage, ATP-producing and utilizing genes in the cercariae as well as stress-response genes in adults have been observed in these stages [61].

As reported by Mbah et al. USPs has actually been observed in different stages of the parasite as it transitions through different environments. The *S. mansoni* G4LZI3 USP has been shown to be expressed in the sporocyst, miracidium and schistosomula stages while the C1M0Q2 USP from the same species is expressed in adult schistosomes [17].

However, we propose that the schistosomula stage is most beneficial for therapeutic interventions using universal stress proteins. This is because up-regulation of these proteins has been observed in this stage, both in *S. mansoni* and *S. japonicum* worms. More so, at this stage, there is a transition from an aquatic environment to a mammalian environment, accompanied with a change in temperature. The parasite has to have the ability to interact with an external environment and also have the skill to adapt to an internal environment. Interestingly, these proteins have not been observed in humans thus making them an interesting and attractive drug target [17]. Secondly, once the cercariae penetrate the human skin to transform into schistosomulae, there should be an adaptation from aerobic surroundings to an anaerobic location. Furthermore, praziquantel has no effect on young schistosomes aged 3–4 weeks post infection but is active against sexually mature adults (6–7 weeks post infection), miracidia and cercariae [31].

Lastly, the UspA family with UspD has been shown in *E. coli* to act as defence mechanisms against superoxide-generating agents [62]. Reactive oxygen species are usually produced under anaerobic conditions and have the potential to oxidize proteins, lipids and nucleic acids, which may eventually lead to enzyme inactivation and genome damage [63]. Nitric oxide and hydrogen peroxide are produced in the mammalian host with the intent of defending against these larval forms by killing them [17]. Under normal circumstances, schistosomulae are more susceptible to oxidative stress and are killed once exposed. However, we suggest that by the action of USPs and through evidence of expression patterns, this parasitic stage escapes or avoids this defence mechanism and goes on to develop into adults, which are able to produce SOD, and GPx antioxidant enzymes that are not found in the miracidia, sporocysts or cercariae [63].

## 6. Recent Advances in Novel Promising Schistosomicides

Because of the risks involved in the continued dependence on praziquantel as the sole chemotherapeutic drug for schistosomiasis, suggestions have been given on what can be used or developed into alternative and promising anti-schistosomal drugs. Thioredoxin reductase and glutathione reductase (which are mammalian detoxifying enzymes), phosphinic amides and oxadiazoles are all enzymes that are proposed to have an effect on schistosomes as inhibitors of the thioredoxin-glutathione reductase schistosome enzyme [24]. Although they are expensive and their mechanism of action is unknown, cyclosporins could also be considered as a new drug alternative due to their ability to kill juvenile schistosome worms as well as the ability to prevent diseases or infections [24]. Cysteine proteases have been considered as chemotherapeutic targets after their activity was shown to be closely linked with hemoglobin digestion and their inhibition shown to disrupt schistosomulae and also reduce egg and worm burden [64]. To avoid toxicity that could result from the use of anti-helminthics, medicinal plants such as garlic, tobacco, pineapple, wormwood and walnut have been suggested as natural anti-helminthic sources [65]. Ali gave an account of different examples of natural products that have promising anti-schistosomal properties [66]. Recently, antimicrobial peptides (AMPs) have come onto the scene as promising alternative anti-schistosomal tools due to their multifunctional properties. It has been suggested that AMPs may play a role against schistosomiasis by acting as immunostimulators, ROS scavengers or through elimination of schistosomulae by selective cytotoxicity [23].

Taken together, since up-regulation of USPs has been detected at the schistosomula stage, and via a variety of mechanisms; the up-regulation in USPs assists the schistosomula to tolerate different environmental and adverse conditions, we propose that selective identification of small molecule inhibitors or peptides as an inhibitor of USPs at this stage could provide a means of targeting schistosomiasis and preventing re-infection of the disease. More research however, has to take place due to the minimal literature on universal stress proteins in schistosomes. However, unveiling of the *S. mansoni*, *S. haematobium* and *S. japonicum* genomes as well as the recent work on the discovery of universal stress proteins in schistosomes could well unfold new opportunities in vaccine development and alternative anti-schistosomal drugs as well as the development of new diagnostic tools.

## 7. Conclusions

In most developing countries, the vicious cycle of poverty and daunting existence of detrimental parasitic infections such as schistosomiasis are some of their biggest challenges. Therefore, addressing poverty should be at the centre of elimination strategies. Intensifying already-in-place interventions such as improving sanitation and health facilities, providing clean and safe water sources, use of molluscicides, as well as continuing with mass treatments using chemotherapeutic drugs are necessary and critical steps to attaining this goal. Praziquantel has been successful so far in decreasing morbidity in endemic areas but mortality rates still soar high and the disease claim thousands of lives every year. Because of its shortcomings, other anti-schistosomal drugs and discovery of vaccines have to be developed as soon as possible. The presence of universal stress proteins in schistosomes presents a very interesting avenue for tackling the problem. Thus, it is believed that future research integrated with understanding social behavior and targeting poverty could abolish this dreadful disease and provide more permanent solutions.

## Figures and Tables

**Table 1:** Universal stress proteins (USPs) found in different living organisms.

Organism/Kingdom	Specie	USP Name	ATP-Binding Potential	References
Bacteria	*Escherichia coli*	UspA	Non ATP-binding	[48,49]
		UspC, UspD	ATP-binding?	
		UspE, UspF, UspG	ATP-binding	
	*Heamophilus influenza*	H10815	Non ATP-binding	[50]
	*Mycobacterium tuberculosis*	RV2623	ATP-binding	[51]
	*Nitrosomonas europea*	NE1028	Non ATP-binding	[52]
	*Salmonella typhimurium*	YdaA	Assumed to bind ATP	[53]
		YnaF	ATP-binding	
	*Thermos thermophiles*	TTHA0350	ATP-binding	[54]
	*Halomonas elongate*	HELO1754	ATP-binding	[55]
Archaea	*Methanococcus jannaschii*	MJ0577	ATP-binding	[56]
	*Archeaoglobus fulgidus*	AF0836	Non ATP-binding	[52]
Plants	*Solanum pennellii*	SpUSP	ATP-binding region	[57]
	*Grosspyiumarbo-retum*	GUSP_1_	Non ATP-binding	[58]
		GUSP_2_		
	*Arabidopsis thaliana*	At3g01520	Suggested to be ATP-binding	[59]
Schistosomes	*S. japonicum*	Q86DX1, Q5DDH7	ATP-binding	[17]
		Q5DED2, Q5DHK1, Q86DW2, Q5DG19, Q5DH64, Q5D136		
	*S. mansoni*	G4V552, G4VPM6, G4LZI3, C1MOQ2, G4VIW9	ATP-binding	[17]

## References

[1] Bergquist R. (2013). Closing in on “perhaps the most dreadful of the remaining plagues”: An independent view of the multidisciplinary alliance to optimize schistosomiasis control in Africa. Acta Trop..

[2] Utzinger J.N., N’Goran E.K., Caffrey C.R., Keiser J. (2011). From innovation to application: Social-ecological context, diagnostics, drugs and intergrated control of schistosomiasis. Acta Trop..

[3] Walker A.J. (2011). Insights into the functional biology of schistosomes. Parasites Vectors.

[4] Gryseels B., Polman K., Clerinx J., Kestens L. (2006). Human scistosomiasis. Lancet.

[5] Lambertucci J.R. (2010). Acute *Schistosomiasis mansoni*: Revisited and reconsidered. Mem. Inst. Oswaldo Cruz.

[6] Hotez P.J., Kamath A. (2009). Neglected Tropical Diseases in sub-Saharan Africa: Review of their prevalence, distribution and disease burden. PLoS Negl. Trop. Dis..

[7] King C.H. (2010). Parasites and poverty: The case of Schistosomiasis. Acta Trop..

[8] Bruun B., Aagaard-Hansen J. (2008). The Social Context of Schistosomiasis and Its Control: An Introduction and Annotated Bibliography.

[9] King C.H., Sturrock R.F., Kariuki H.C., Hamburger J. (2006). Transmission control for schistosomiasis—Why it matters now. Trends Parasitol..

[10] Colley D.G., Bustinduy A.L., Secor W.E., King C.H. (2014). Human Schistosomiasis. Lancet.

[11] Elbaz T., Esmat G. (2013). Hepatic and intestinal schistosomiasis: Review. J. Adv. Res..

[12] Chitsulo L., Engels D., Montresor A., Savioli L. (2000). The global status of schistosomiasis and its control. Acta Trop..

[13] Rollinson D., Knopp S., Levitz S., Stothard J.R., Tchuenté L.A.T., Garba A., Mohammed K.A., Schur N., Person B., Colley D.G. (2013). Time to set the agenda for schistosomiasis elimination. Acta Trop..

[14] Steinmann P., Keiser J., Bos R., Tanner M., Utzinger J. (2006). Schistosomiasis and water resources development: Systematic review, meta-analysis and estimates of people at risk. Lancet Infect. Dis..

[15] Berry A., Moné H., Iriart X., Mouahid G., Aboo O., Boissier J., Fillaux J., Cassaing S., Debuisson C., Valentin A. (2014). Schistosomiasis, Haematobium, Corsica, France. Emerg. Infect. Dis..

[16] Knight M., Elhelo O., Smith M., Haugen B., Miller A., Raghavan N., Wellman C., Cousin C., Dixon F., Mann V. (2015). Susceptibility of Snails to Infection with Schistosomes is influenced by Temperature and Expression of Heat Shock Proteins. Epidemiology.

[17] Mbah A.N., Mahmud O., Awofolu O.R., Isokpehi R.D. (2013). Inferences on the biochemical and environmental regulation of universal stress proteins from Schistosomiasis parasites. Adv. Appl. Bioinform. Chem..

[18] Conlon C.P. (2005). Schistosomiasis. Medicine.

[19] Coon D.R. (2005). Schistosomiasis: Overview of the history, biology, clinicopathology and laboratory diagnosis. Clin. Microbiol. Newslett..

[20] Aragon A.D., Imani R.A., Blockburn V.R., Cunningham C. (2008). Microarray based analysis of temperature and oxidative stress induced messenger RNA in *Schistosoma mansoni*. Mol. Biochem. Parasitol..

[21] Bica I., Hamer D.H., Stadecker M.J. (2000). Hepatic schistosomiasis. Infect. Dis. Clin. N. Am..

[22] Adenowo A.F., Oyinloye B.E., Ogunyika B.I., Kappo A.P. (2015). Impact of human schistososmiasis in sub-Saharan Africa. Braz. J. Infect. Dis..

[23] Oyinloye B., Adenowo F., Gxaba N., Kappo A. (2014). The promise of antimicrobial peptides for treatment of human schistosomiasis. Curr. Drug Targets.

[24] Doenhoff M.J., Cioli D., Utzinger J. (2008). Praziquantel: Mechanisms of action, resistance and new derivatives for schistosomiasis. Curr. Opin. Infect. Dis..

[25] Chevalier F.D., Le Clec’h W., Eng N., Rugel A.R., de Assis R.R., Oliveira G., Holloway S.P., Cao X., Hart P.J., LoVerde P.T. (2016). Independent origins of loss-of-function mutations conferring oxamniquine resistance in a Brazilian schistosome population. Int. J. Parasitol..

[26] Aragon A.D., Imani R.A., Blackburn V.R., Cupit P.M., Melman S.D., Goronga T., Webb T., Loker E.S., Cunningham C. (2009). Towards an understanding of the mechanism of action of Praziquantel. Mol. Biochem. Parasitol..

[27] Fallon P.G., Tao L.-F., Ismail M.M., Bennete J.L. (1996). Schistosome resistance to Praziquantel: Fact or artefact?. Parasitol. Today.

[28] Alsaqabi S.M., Lotfy W.M. (2014). Praziquantel. J. Vet. Sci. Technol..

[29] Cioli D., Pica-Mattoccia L., Basso A., Guidi A. (2014). Schistosomiasis control: Praziquantel forever?. Mol. Biochem. Parasitol..

[30] Caffrey C.R. (2007). Chemotherapy of Schistosomiasis: Present and future. Curr. Opin. Chem. Biol..

[31] Greenberg R.M. (2005). Are Ca^2+^ channels targets of Praziquantel action?. Int. J. Parasitol..

[32] Melman S.D., Steinauer M.L., Cunningham C., Kubatko L.S., Mwangi I.N., Wynn N.B., Mutuku M.W., Karanja D.M.S., Colley D.G., Black C.L. (2009). Reduced susceptibility to Praziquantel among naturally occurring Kenyan isolates of *Schistosoma mansoni*. PLoS Negl. Trop. Dis..

[33] William S., Botros S., Ismail M., Farghally A., Day T.A., Bennett J.L. (2001). Praziquantel-induced tegumental damage in vitro is diminished in schistosomes derived from praziquantel-resistant infections. Parasitology.

[34] Doenhoff M.J., Kusel J.R., Coles G.C., Cioli D. (2002). Resistance of *Schistosoma mansoni* to praziquantel: Is there a problem?. Trans. R. Trop. Med. Hyg..

[35] Coeli R., Baba E.H., Araujo N., Coelho P.M.Z., Oliveira G. (2012). Praziquantel Treatment Decreases *Schistosoma mansoni* Genetic Diversity in Experimental Infections. PLoS Negl. Trop. Dis..

[36] Couto F.F.B., Coelho P.M.Z., Araújo N., Kusel J.R., Katz N., Jannotti-Passos L.K., Mattos A.C.A. (2011). *Schistosoma mansoni*: A method for inducing resistance to praziquantel using infected *Biomphalaria glabrata* snails. Mem. Inst. Oswaldo Cruz.

[37] McCreesh N., Boot M. (2014). The effect of increasing water temperatures of *Schistosoma mansoni* transmission and *Biomphalaria pfeifferi* population dynamics: An agent based modellign study. PLoS ONE.

[38] Walz Y., Wegmann M., Decg S., Raso G., Utzinger J. (2015). Risk profiling of schistosomiasis using remote sensing: Approaches, challenges and outlook. Parasites Vectors.

[39] Upathum E.S. (1973). Location of *Biomphalaria glabrata* (SAY) by Miracidia of *Schistosoma mansoni* Sambon in Natural Standing and Running Waters of the West Indian Island of St Lucia. Int. J. Parasitol..

[40] Negrão-Corrêa D., Pereira C.A.J., Rosa F.M., Martins-Souza R.L., Andrade Z.A., Coelho P.M.Z. (2007). Molluscan response to parasite *Biomphalaria* and *Schistosoma mansoni* interaction. ISJ.

[41] Negrão-Corrêa D., Mattos A.C.A., Pereira C.A.J., Martins-Souza R.L., Coelho P.M.Z. (2012). Interaction of *Schistosoma mansoni* Sporocysts and Hematocytes of *Biomphalaria*. J. Parasitol. Res..

[42] Raghavan N., Miller A.N., Gardener M., FitzGerald P.C., Kerlavage A.R., Johnston D.A., Lewis F.A., Knight M. (2003). Comparative gene analysis of *Biomphalaria glabrata* hemocytes pre- and post-exposure to miracidia of *Schistosoma mansoni*. Mol. Biochem. Parasitol..

[43] Theron A., Rognon A., Gourbal B., Mitta G. (2014). Multi-parasite host susceptibility and multi-host parasite infectivity: A new approach of the *Biomphalaria glabrata/Schistosoma mansoni* compatibility polymorphism. Infect. Genet. Evol..

[44] LoVerde P.T. (1998). Do antioxidants play a role in Schistosome hot-parasite interactions?. Parasitol. Today.

[45] Pearce E.J., MacDonald A.S. (2002). The Immunobiology of Schistosomes. Nat. Rev. Immunol..

[46] Jenkins S.J., Hewitson J.P., Jenkins G.R., Mountford A.P. (2005). Modulation of the host’s immune response by schistosome larvae. Parasite Immunol..

[47] Colley D.G., Secor W.E. (2014). Immunology of human schistosomiasis. Parasite Immunol..

[48] Kvint K., Nachin L., Diez A., Nyström T. (2003). The bacterial universal stress protein: Function and regulation. Curr. Opin. Microbiol..

[49] Forêt S., Seneca F., de Jong O., Bieller A., Hemmich G., Augustin R., Hayward D.C., Ball E.E., Bosch T.C.G., Agata K. (2011). Phylogenomics reveals an anomalous distribution of USP genes in metazoans. Mol. Biol. Evol..

[50] Sousa M.C., McKay D.B. (2001). Structure of the universal stress protein of *Haemophilus influenzae*. Structure.

[51] Drumm J.E., Mi K., Bilder P., Sun M., Lim J., Bielefeldt-Ohmann H., Basaraba R., Therefore M., Zhu G., Tufariello J.M. (2009). *Mycobacterium tuberculosis* universal stress protein Rv2623 regulates bacillary growth by ATP-Binding: Requirement for establishing chronic persistent infection. PLoS Pathog..

[52] Tkaczuk K.L., Shumilin A., Chruszcz I., Evdokimova E., Savchenko A., Minor W. (2013). Structural and functional insight into the universal stress protein family. Evol. Appl..

[53] Bangera M., Panigrahi R., Sagurthi R., Savithri H.S., Murthy M.R.N. (2015). Structural and functional analysis of two universal stress proteins YdaA and YnaF from Salmonella typhimurium: Possible roles in microbial stress tolerance. J. Struct. Biol..

[54] Lino H., Shimizu N., Goto M., Ebihara A., Fukui K., Hirotsu K., Kuramitsu S. (2011). Crystal structure of the tandem-type universal stress protein TTHA0350 from *Thermus thermophiles* HB8. J. Biochem..

[55] Schweikhard E.S., Kuhlmann S.I., Kunte H.J., Grammann K., Ziegler C.M. (2010). Structure and function of the universal stress protein TeaD and its role in regulating the ectoine transporter TeaABC of *Halomonas elongata* DSM2581T. Biochemistry.

[56] Zarembinski T.I., Hung L.W., Mueller-Dieckmann H.J., Kim K.K., Yokota H., Kim R., Kim S.H. (1998). Structure-based assignment of the biochemical function of a hypothetical protein: A test case of structural genomics. Proc. Natl. Acad. Sci. USA.

[57] Loukehaich R., Wang T., Ouyang B., Ziaf K., Li H., Zhang J., Lu Y., Ye Z. (2012). SpUSP, an annexin-interacting universal stress protein, enhances drought tolerance in tomato. J. Exp. Bot..

[58] Maqbool A., Zahur M., Husnain T., Riazuddin S. (2009). GUSP1 and GUSP2, two drought-responsive genes in *Gossypium arboreum* have homology to universal stress proteins. Plant Mol. Biol..

[59] Kim D.J., Bitto E., Bingman C.A., Kim H.J., Han B.W., Phillips G.N. (2015). Crystal structure of the protein At3g01520, a eukaryotic universal stress protein-like protein from *Arabidopsis thaliana* in complex with AMP. Proteins.

[60] Isokpehi R.D., Mahmud O., Mbah A.N., Simmons S.S., Avelar L., Rajnarayanan R.V., Udensi U.K., Ayensu W.K., Cohly H.H., Brown S.D. (2011). Developmental regulation of genes encoding universal stress proteins in *Schistosoma mansoni*. Gene Regul. Syst. Biol..

[61] Jolly E.R., Chin C., Miller S., Bahgat M.M., Lim K.C., DeRisi J., McKerrow J.H. (2007). Gene expression patterns during adaptation of a helminth parasite to different environmental niches. Genome Biol..

[62] Nachin L., Nannmark U., Nystrom T. (2005). Differential roles of the universal stress proteins of *Escherichia coli* in oxidative stress resistance, adhesion and motility. J. Bacteriol..

[63] Chiumiento L., Bruschi F. (2009). Enzymatic antioxidant systems in helminth parasites. Parasitol. Res..

[64] Ribeiro-dos-Santos G., Verjovski-Ameida S., Leite L.C.C. (2006). Schistosomiasis-a century searching for chemotherapeutic drugs. Parasitol. Res..

[65] Yadav P., Singh R. (2011). A review on anthelminthic drugs and their future scope. Int. J. Pharm. Pharm. Sci..

[66] Ali S.A. (2011). Natural products as therapeutic agents for schistosomiasis. Res. J. Med. Plants.

